# Aerosols cause intraseasonal short-term suppression of Indian monsoon rainfall

**DOI:** 10.1038/s41598-017-17599-1

**Published:** 2017-12-11

**Authors:** Prashant Dave, Mani Bhushan, Chandra Venkataraman

**Affiliations:** 1 Interdisciplinary Programme in Climate Studies, Indian Institute of Technology Bombay, Mumbai, 400076 India; 2 Department of Chemical Engineering, Indian Institute of Technology Bombay, Mumbai, 400076 India

## Abstract

Aerosol abundance over South Asia during the summer monsoon season, includes dust and sea-salt, as well as, anthropogenic pollution particles. Using observations during 2000–2009, here we uncover repeated short-term rainfall suppression caused by coincident aerosols, acting through atmospheric stabilization, reduction in convection and increased moisture divergence, leading to the aggravation of monsoon break conditions. In high aerosol-low rainfall regions extending across India, both in deficient and normal monsoon years, enhancements in aerosols levels, estimated as aerosol optical depth and absorbing aerosol index, acted to suppress daily rainfall anomaly, several times in a season, with lags of a few days. A higher frequency of prolonged rainfall breaks, longer than seven days, occurred in these regions. Previous studies point to monsoon rainfall weakening linked to an asymmetric inter-hemispheric energy balance change attributed to aerosols, and short-term rainfall enhancement from radiative effects of aerosols. In contrast, this study uncovers intraseasonal short-term rainfall suppression, from coincident aerosol forcing over the monsoon region, leading to aggravation of monsoon break spells. Prolonged and intense breaks in the monsoon in India are associated with rainfall deficits, which have been linked to reduced food grain production in the latter half of the twentieth century.

## Introduction

The Indian summer monsoon affects water availability and therefore water management related to rain-fed agricultural practices^1,2^. Long-term changes in Indian monsoon precipitation that are linked to aerosol radiative forcing, termed slow-responses, have been caused by thermodynamic adjustments of mean temperature and moisture content^3,4^, reductions in land–sea temperature differences and zonal winds, and dynamic circulation adjustments to regional energy imbalances^5–7^. These, in turn, have been associated with precipitation deficits on multi-decadal time scales. Short-term changes in Indian monsoon precipitation that are linked to aerosols, termed fast-responses, occur through the enhancement of meridional surface temperature or pressure gradients^6,8^ and mid-tropospheric diabatic heating^9^, which cause an increase in the northward transport of moisture, leading to the onset and enhancement of precipitation on time scales of days to a month.

The importance of synoptic, large-scale convection in supporting vertically integrated moisture transport in monsoon systems is well established^4,10,11^. Aerosols were linked to significant decreases in convective instability over India, inferred from modeled lower atmosphere warming^4^ and increased tropospheric temperature trends, in agreement with microwave sounder measurements during 1979–2003. In studies not specifically related to the Indian monsoon, aerosols have been linked to the inhibition of cloud and precipitation development, by altering the vertical profile of heating rate, inducing stabilization^12^ and suppressing mesoscale convective motion.

Aerosol abundance is persistent over the Indian subcontinent during the summer monsoon season^13–15^. Aerosol-induced effects on cloud microphysical properties have been linked to both precipitation suppression^16^ and invigoration^17^, at different aerosol levels. These occur through changes in cloud droplet size distributions, redistribution of precipitable water, and latent heat changes associated with condensation and evaporation^12^. Reduction in the median size and width of cloud droplet distributions reduces the efficiency of droplet growth^18^. Fast microphysical effects have been linked to precipitation shut-off in ship tracks for pristine marine clouds^19^. Recently, influences of aerosols in the monsoon region have been reported, suggestive of inhibition or invigoration of clouds and rainfall^20,21^.

To our knowledge, the causal effects of coincident aerosols on the changes in Indian monsoon precipitation have not been investigated through observational analysis. Here we find causal relationships between aerosol enhancement and suppression of lagged daily precipitation and mean cloud drop sizes, through atmospheric stabilization, increased moisture divergence and reduced convection, leading to a higher frequency of prolonged rainfall breaks.

## Results

Aerosol and cloud properties from satellite observations^22–24^, precipitation from ground based measurements^25^, and meteorological variables from European Centre for Medium-Range Weather Forecasts (ECMWF) re-analysis (ERA)-interim reanalysis data^26^ from 2000–2009 over the Indian subcontinent (6.5–40°N and 66.5–100°E), at 1 × 1° resolution for the monsoon months of June to September (JJAS), were used for the analysis (for more information see Methods). Precipitation data used were from 1803 irregularly located meteorological stations over India^25^, reported to be gridded to 1 × 1^o^, using Shepard’s interpolation methodology, yielding approximately 350 pixels over the Indian domain. Normalized daily anomaly was calculated for each variable, as deviation for a specific day and pixel from its mean (calculated across years), normalized by its standard deviation (for more information see Methods). The data were clustered for each year using hierarchical clustering^27^, using season average of normalized anomaly of AOD and precipitation, to identify clusters of (a) high AOD-low precipitation (HL), (b) low AOD-low precipitation (LL), (c) high AOD-high precipitation (HH), and (d) low AOD-high precipitation (LH). Cluster average time series of normalized anomalies of AOD and precipitation were used to detect Granger causality^28^ which tests statistically significant improvement in the prediction of precipitation, using past information of AOD, as compared to only past information of precipitation (for more information see Methods). In case of causal association, path analysis^29^ was used to enable identification of mechanisms through which AOD influenced precipitation (for more information see Methods).

### Causal Influence of Aerosols on Short-term Precipitation Suppression

**Figure 1:**
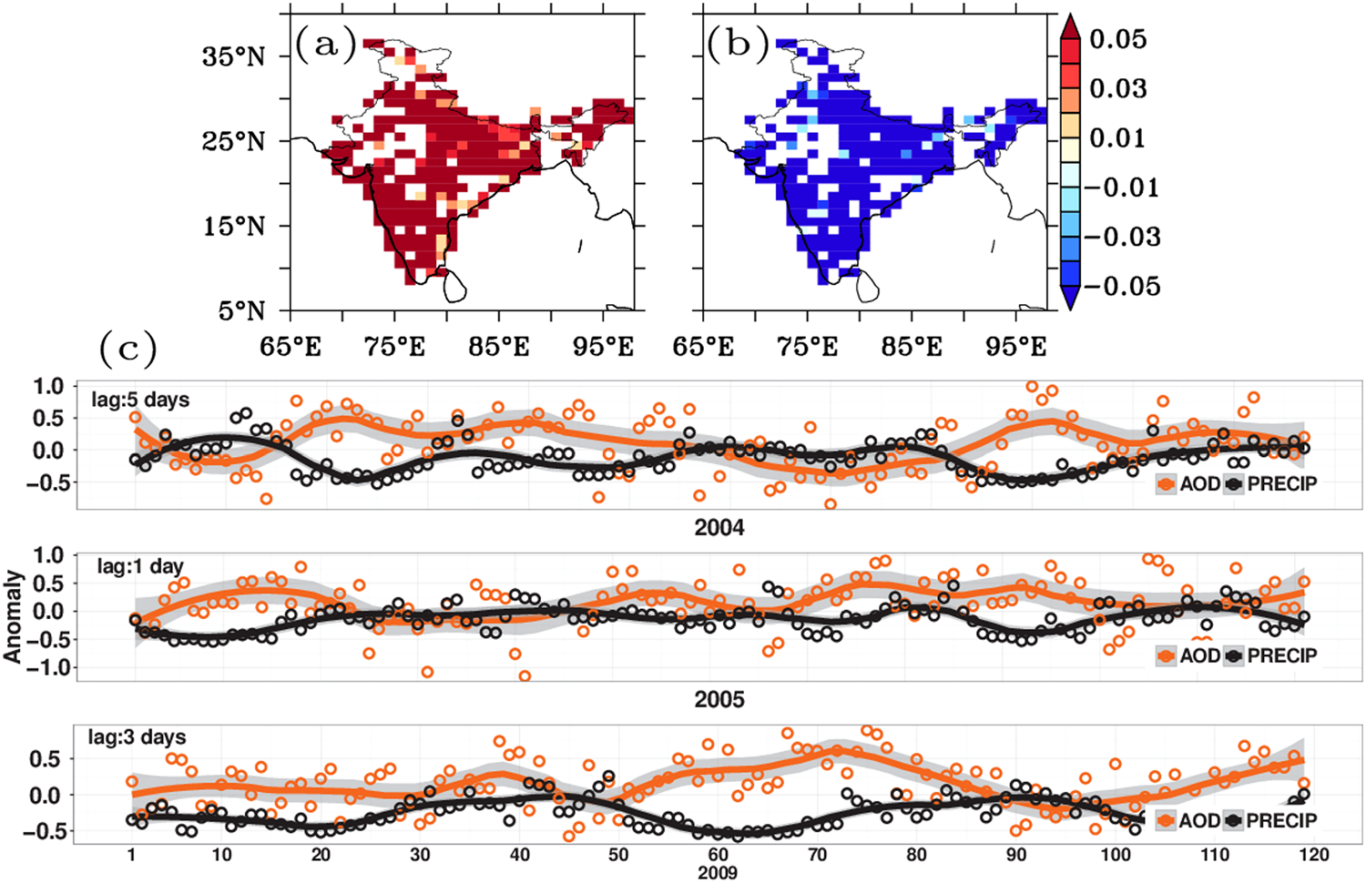
HL cluster pixel distribution and co-variability of AOD-Precipitation: Spatial distribution of pixels with high AOD and low precipitation anomaly along with cluster averaged temporal series for individual years: (**a**) and (**b**) Seasonal averaged spatial distribution of HL overlapping clusters for years 2004, 2005 and 2009 for AOD and precipitation respectively. (**c**) Cluster averaged time series over HL cluster (precipitation shifted by lag corresponding to maximum correlation magnitude). The composite plot of pixels shows that the high AOD-low precipitation covers wide area across the country. In the time series plot between AOD and precipitation anomaly, precipitation anomaly was shifted by the lag corresponding to maximum correlation coefficient magnitude. Multiple instances of enhanced AOD associated with suppressed precipitation can be seen in Fig. 1(c). Figure was created using R statistical tool v3.3.1 (https://www.r-project.org/) and FERRET v7.0 (http://www.ferret.noaa.gov/Ferret/).

It was found that high aerosol-low precipitation (HL) clusters extended over large parts of India in 2004, 2005 and 2009 (Supplementary Fig. 2). In HL clusters, a leading positive AOD anomaly caused a negative precipitation anomaly, with a lag time of 1–5 days, during the JJAS monsoon season in 2004, 2005, and 2009 (Fig. 1a,b). However, no causal influence was found in the corresponding LL clusters, characterized by low aerosol abundance (Supplementary Figs. 3,4). Precipitation suppression lagged aerosol enhancement and lasted one to five days (2004, 2–5 days; 2005, 1–2 days; 2009, 2–5 days), with the maximum influence, in terms of correlation coefficient magnitude, occurring on different days (2004, day 5; 2005, day 1; 2009, day 3). The corresponding lagged time series between AOD anomaly and precipitation anomaly (Fig. 1c), corresponding to maximum correlation magnitude, showed several intra-seasonal periods of high AOD anomaly followed by periods of low precipitation. These negative causal relationships did not manifest in 2000, 2001, and 2002; 2006 and 2008 lacked sufficient data (<50 pixels in HL and LL clusters) for conclusive results. A positive causal effect of daily AOD on daily precipitation was found in 2003 and 2007, identified as abundant monsoon years^30^.

In addition to total aerosol abundance (measured by AOD), the effects of absorbing aerosols (measured by absorbing aerosol index, AAI) were examined in the HL and LL clusters. Again a positive anomaly in AAI exerted a strong causal influence on lagged negative precipitation anomaly (2004 and 2005, 2–5 days; 2009, 1 day), which lasted one to five days, with strongest causal influence occurring on different days (quantified as correlation coefficient) in different years (2004, day 5; 2005, day 5; 2009, day 1). This behavior also manifested in LL clusters (with negative AOD anomalies) in 2005 and 2009. Since clustering was performed with AOD, the LL clusters contained many (30–40%) positive AAI anomaly values and thus included several days of high absorbing aerosol levels. Overall, enhanced levels of aerosols caused suppression of lagged daily precipitation, 3–5 times during the monsoon season, in high aerosol-low precipitation regions (HL).

Other studies found positive correlations between AOD and cloud properties^20^ and daily precipitation over the monsoon region^31^, suggesting a cloud invigoration effect, however, did not test for causation. The aerosol-cloud invigoration effect is beyond the scope of the present study, which focuses only on variables linked to aerosol-induced suppression of precipitation.

### Cause–Effect Model Development and Validation

The physical mechanisms (causality) underlying the observed aerosol caused suppression of precipitation are studied using cause-effect model and path-analysis.

To unravel the mechanism of AOD- or AAI-induced precipitation suppression, it was postulated that a microphysical pathway linked AOD with precipitation through the mean cloud droplet effective radius (CDER) (AOD–CDER–PRECIP), while a radiative pathway linked aerosols (AOD or AAI) with precipitation through the lapse rate (defined as AOD–lapse rate–PRECIP and AAI–lapse rate–PRECIP). Causality was first tested between AOD and CDER, AOD and lapse rate, and AAI and lapse rate, and path analysis was used to segregate and quantify the effects of the two mediating pathways. These pathways were compared with pathways that directly linked column water vapor (CWV) with both precipitation (PRECIP) and CDER, to evaluate their respective strengths^32^.

**Figure 2:**
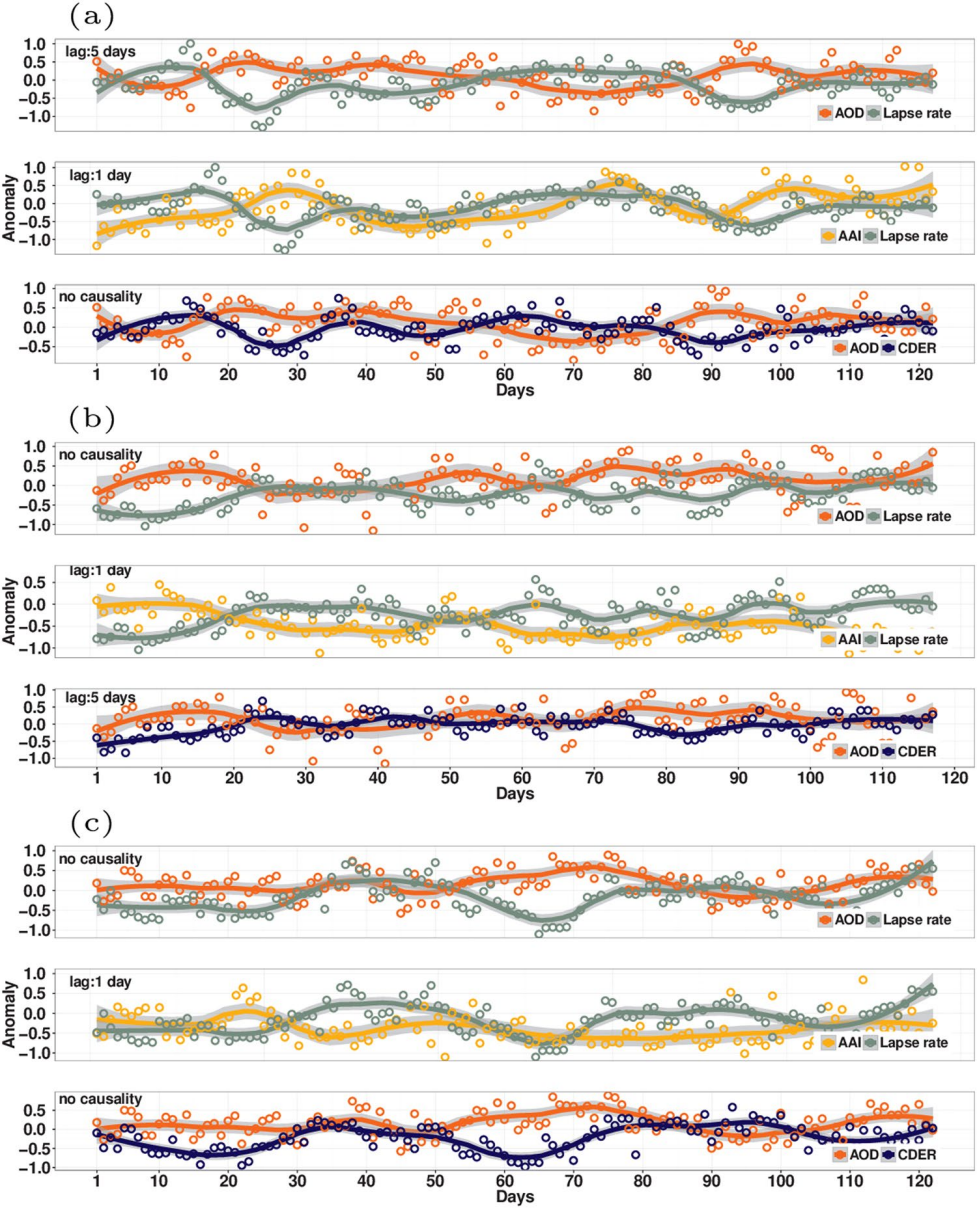
Co-variation of aerosols: AOD-lapse rate (lapse rate shifted by lag corresponding to maximum correlation magnitude), AAI-lapse rate (lapse rate shifted by lag corresponding to maximum correlation magnitude) and AOD-cloud droplet effective radius (cloud droplet effective radius shifted by lag corresponding to maximum correlation magnitude) time series plots, for years (**a**) 2004, (**b**) 2005 and (**c**) 2009, in HL cluster. Similar to Fig. 1(c), here multiple instances of enhanced AOD anomaly associated with suppressed cloud droplet size and suppressed lapse rate can be seen. The AAI anomaly enhancement with suppression of lapse rate can also be seen in the current figure. Figure was created using R statistical tool v3.3.1 (https://www.r-project.org/).

In high aerosol-low precipitation (HL) regions, enhancement of AOD caused a reduction in lapse rate (2004) and in CDER (2005), extending to five days (panels 1 and 3, Fig. 2a,b). No significant effect of AOD on CDER was found in 2004 or 2009. Here we found aerosol induced increase in static stability and decrease in moisture availability, with short time-lags of 1-5 days, subsequently, causing reduction in cloud droplet size (CDER). This effect of reduction in CDER via divergence of moisture takes place over a period of days as compared to microphysical effects, where increases in aerosols within clouds leads to formation of larger number of smaller drops on time scales of minutes to hours. Enhancement of AAI exerted suppression of lapse rate (stabilization) at shorter lag times of one day (panel 2, Fig. 2a,c), indicating that the radiative effects on lapse rate changes were largely influenced by absorbing aerosols in all three years. In contrast, no causal influences were found in low aerosol-low precipitation (LL) regions, implying absence of these effects.

### Cloud Microphysical Pathway

**Figure 3:**
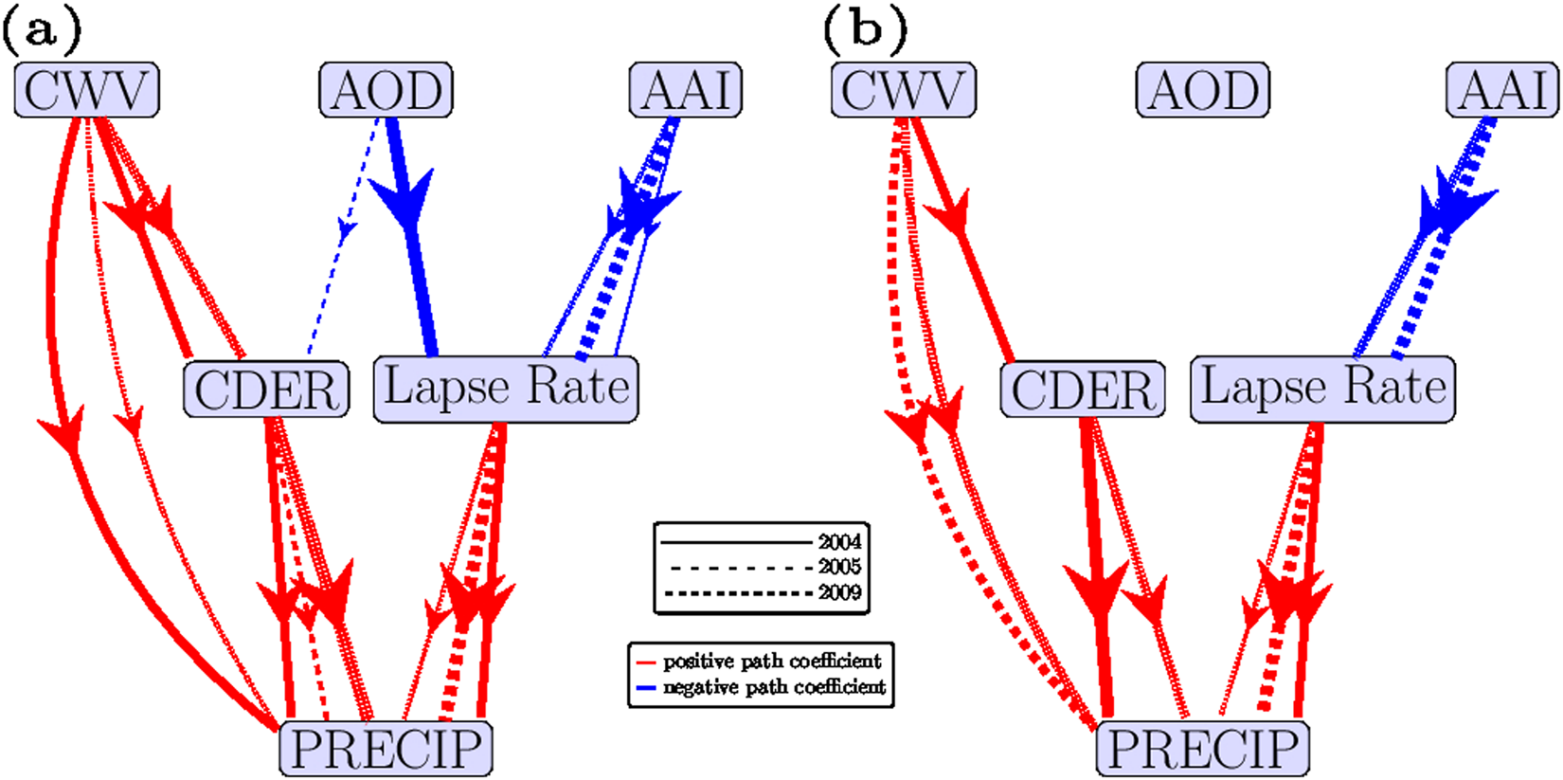
Path diagram: Cause-effect model for 2004, 2005 and 2009: (**a**) HL (**b**) LL (line-width represents absolute magnitude of path coefficient, larger width implying greater absolute path-coefficient). Arrows represent the direction of causal influence, and color indicates the sign of causal influence, with red showing positive, blue showing negative, and different line styles representing different years.

The cause-effect model and lagged correlation coefficients were used as input in the path analysis; the overall correlation coefficients were segregated into path coefficients whose sign and magnitude indicated the direction and strength of the causal influence (Fig. 3a,b). The cloud microphysical pathway (Fig. 3a) showed inverse effects of AOD on CDER (blue color; arrow direction) or mean drop size, but direct effects of CDER on PRECIP (red color; arrow direction), indicating increases in AOD causing lagged decreases in CDER and rainfall. A positive causal influence of water vapor availability (CWV) on both PRECIP and CDER showed the expected relation of increased moisture availability to cloud and rainfall development. The CWV–PRECIP direct positive pathway was significantly stronger than the negative AOD–CDER–PRECIP pathway, based on the magnitudes of the overall path effects (Supplementary Table 1), indicating that rainfall suppression through changes in cloud drop size was not a strong pathway. In the low aerosol-low precipitation (LL) regions no causal influence of aerosol level on CDER was found (missing causal lines between AOD and CDER in Fig. 3b), however, moisture availability exerted positive causal effects on rainfall, both directly (CWV–PRECIP, Fig. 3b) and indirectly through CDER (CWV–CDER–PRECIP). Thus, the aerosol effects acted only in the high-aerosol regions, but moisture effects acted in both high and low aerosol regions.

Cloud microphysical processes, which influence rainfall suppression, result from direct increases in aerosols at cloud level, leading to the redistribution of moisture to a larger number of smaller drops. This reduces coalescence efficiency, slowing down the conversion of cloud drops to raindrops or graupel^18^. Raindrop formation is initiated from vapor condensation and collision/coalescence processes, on time scales of a minute, but it subsequently intensifies from scavenging of small cloud drops by gravitational settling of larger drops (termed autoconversion). This leads to precipitation onset on time scales of about 15–20 minutes^33^. Such fast microphysical effects have been linked to precipitation shut-off in ship tracks in pristine marine clouds^19^. Microphysical and radiative processes are largely independent, occurring at different ranges of AOD values; the time responses of the microphysical processes are much shorter than those of the radiative processes^34^. The presence of 1–5 day lag times between aerosol enhancement and CDER or precipitation suppression indicates that these causal relationships might not be a direct microphysical effect, which typically acts on time scales of minutes to hours.

### Radiative Pathway

In regions of high-aerosol and low-precipitation, the radiative pathway (Fig. 3a) showed inverse effects of AOD on lapse rate (blue color; arrow direction) calculated as the slope of potential temperature, with a lower magnitude of lapse rate, indicating higher atmospheric stability. Positive effects of lapse rate on PRECIP (red color; arrow direction), indicated increases in AOD causing lagged decreases in lapse rate and rainfall. The causal influence between AAI and lapse rate was especially strong in 2004, 2005 and 2009, substantiated by larger magnitudes of negative path coefficients (Supplementary Table 1), compared to that of AOD which acted only in 2004. The radiative pathway of absorbing aerosols (AAI–lapse rate–PRECIP), equaled the moisture-rainfall (CWV-PRECIP) effect in strength in some years, indicating the potential for strong aerosol-induced rainfall suppression. A stronger influence of the radiative pathway, than the cloud microphysical pathway, was found (larger negative values of path coefficients; Supplementary Table 1) on rainfall suppression. The radiative pathway showed significant effects even in the low-aerosol regions (LL clusters which were based on AOD) indicating overall more spatially widespread effects of aerosols on rainfall suppression. The radiative and cloud microphysical pathways, along with CWV, together explained approximately half of total precipitation variability (PRECIP *R* ^2^ > 0.50; Supplementary Table 1). Exclusion of the radiative pathway from the model resulted in a significant drop in PRECIP *R* ^2^ for the HL and LL clusters (Supplementary Table 2), highlighting the strong effects of aerosol-induced atmospheric stabilization on precipitation suppression. The distributions of anomaly values of CWV and cloud fraction (CF), which could influence rainfall development, were not statistically different (Supplementary Fig. 5a,b) between the high- and low-aerosol regions, further supporting aerosol-induced stabilization as the primary cause of the observed rainfall suppression.

Short-term radiative effects can manifest within a day of an increase in aerosol levels and last for two or more days^35–38^. The absorption of solar radiation by aerosols has been linked to local atmospheric heating, with cooling of the surface leading to a reduction in the atmospheric lapse rate or a consequent increase in stability^36,39^; this is consistent with the radiative pathway seen in this study. The effect is further linked to a suppression of moisture and heat fluxes from the surface^34^, a reduction of convection^12^, and the vertical mixing of moisture. Aerosol levels were linked to a reduction in cloud fraction and drop sizes of shallow continental clouds^34,40^. Higher atmospheric stabilization by aerosols was linked to modeled decreases in monsoon precipitation on multi-decadal time scales^4^, while it was suggested as significant on short time scales in another study^5^. An increase in black carbon aerosols increased stability of the boundary layer and reduced convection, which further reduced cumulus precipitation in large eddy simulations (LES)^12^ and general circulation model simulations^41^. To our knowledge, the findings here are the first demonstration of causality of aerosol-induced atmospheric stabilization on Indian monsoon precipitation suppression.

In contrast with the aerosol-induced suppression of precipitation found in this study, Sarangi *et al*.^31^ found an increase in daily precipitation intensity with increased aerosol loading over the core monsoon zone. Differences in the studies include the use of clustering into HL and LL regions here, in contrast with the use of non-segregated data in the other study. Further, cloud fraction in the present study ranged from 0.5 to 1 in the HL and 0.3 to 1 in the LL regions, in contrast with larger cloud fractions considered in the other study. Aerosol-induced invigoration of rainfall acts at different levels of AOD on different regimes of cloud fraction, in comparison with the suppression mechanism investigated here, and manifests in changes in micro- and macro-physical cloud properties, the analysis of which is beyond the scope of the present study.

### Mechanisms of Short-term Precipitation Suppression

Further analysis was performed to link the observed causality between enhanced aerosol levels and short-term precipitation suppression to factors typically used to explain monsoon variability, such as, vertical integral of divergence of moisture flux (VIDMF), vertical wind (ω850) and surface pressure^10,42,43^. Increases in VIDMF and ω850 have been linked to suppression in precipitation, while an increase in surface pressure has been associated with short monsoon break spells^44^.

**Figure 4:**
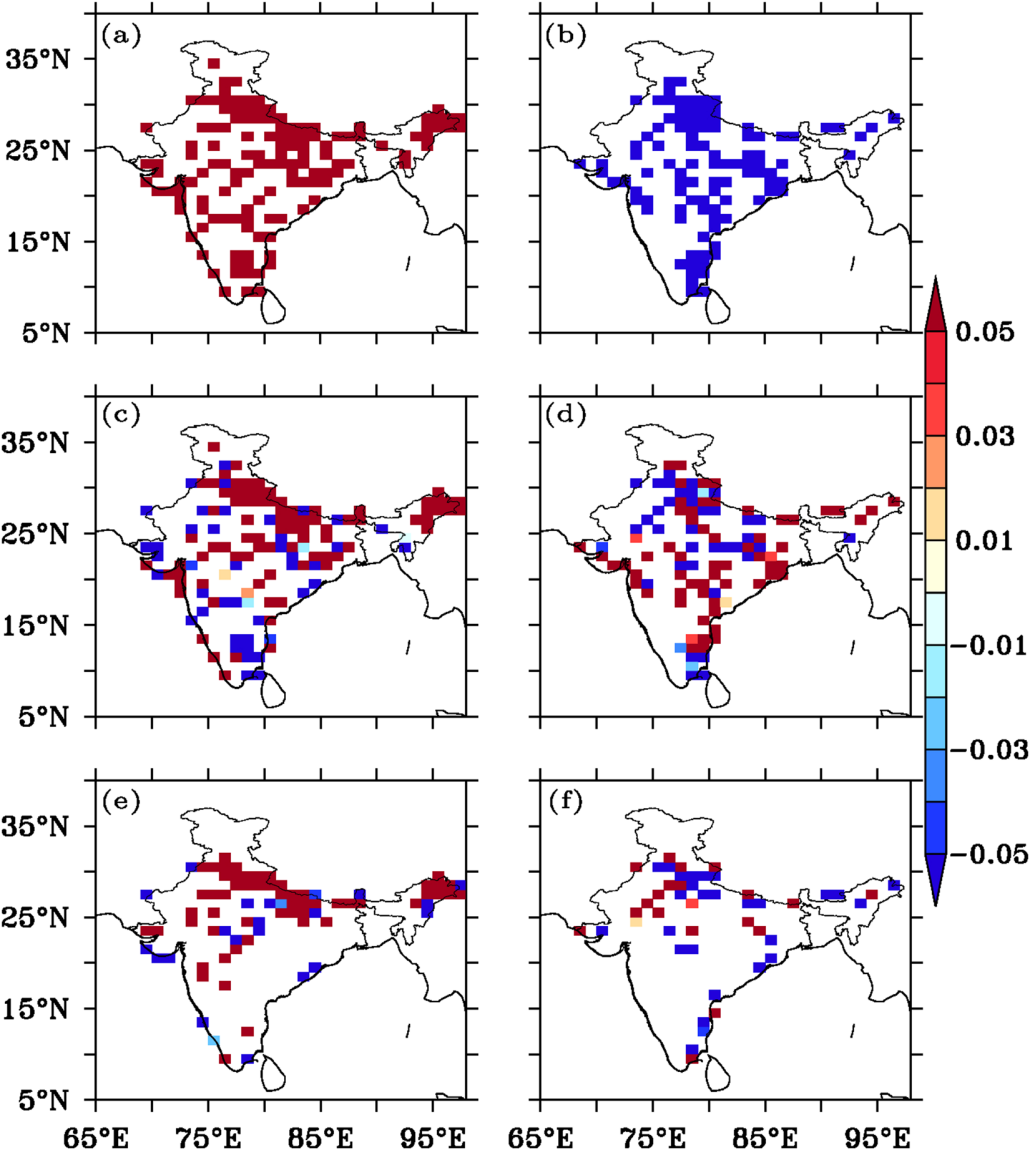
VIDMF and ω850 anomaly variation with AOD anomaly: VIDMF anomaly and ω850 anomaly composite with varying AOD anomaly threshold. (**a**) AOD anomaly >0.7, (**b**) AOD anomaly <−0.7 (**c**) VIDMF anomaly (AOD anomaly >0.7), (**d**) VIDMF anomaly (AOD anomaly <−0.7) (**e**) ω850 anomaly (AOD anomaly >0.7) and (**f**) ω850 anomaly (AOD anomaly <−0.7). It can be seen that high AOD anomaly (>0.7) is associated with more divergence of moisture and downward wind as compared to low AOD anomaly (<−0.7). Figure was created using FERRET v7.0 (http://www.ferret.noaa.gov/Ferret/).

In periods of high aerosol levels (AOD anomaly >0.7 for at least three consecutive days) in the high-aerosol regions, VIDMF and ω850 anomalies (upward wind being negative) were found to be positive (Fig. 4a,c,e), indicating higher moisture flux divergence and a simultaneous reduction in convective activity in the column, in contrast to that in low aerosol periods (Fig. 4b,d,f). These effects were more pronounced during periods of higher aerosol levels (at different AOD anomaly thresholds; Supplementary Fig. 6) from an increase in gross moist stability, defined as the ratio of vertically integrated horizontal divergence of moisture to vertical convection, consistent with earlier studies^45^, associated with subsequently reduced precipitation.

The role of common moderating meteorological variables is often debated in relation to aerosol–cloud–precipitation interactions. Surface pressure is typically identified as one of these variables, whose positive anomalies have been linked to break spells in monsoon precipitation^44^. Increases in AOD anomalies had causal effects on increases in anomalies of surface pressure and VIDMF, with lags of 1–5 days (Supplementary Table 3). In HL regions, a greater frequency of positive surface pressure anomalies was found (Supplementary Fig. 7). However, there was no causality between increased anomalies in surface pressure and those in VIDMF (Supplementary Table 3), ruling out the role of surface pressure in the observed effects.

The monsoon region is reported to experience the persistence of both dust aerosols^13,46^ and build-up of anthropogenic fine particles^47–49^, the combined effects of which can diminish surface reaching radiation and can cool the surface, subsequently increasing the atmospheric stability. Further, transient occurrence of black carbon aerosols has been measured both at surface and at elevations of 1 to 3 km over India and adjoining oceans^50,51^.

**Figure 5:**
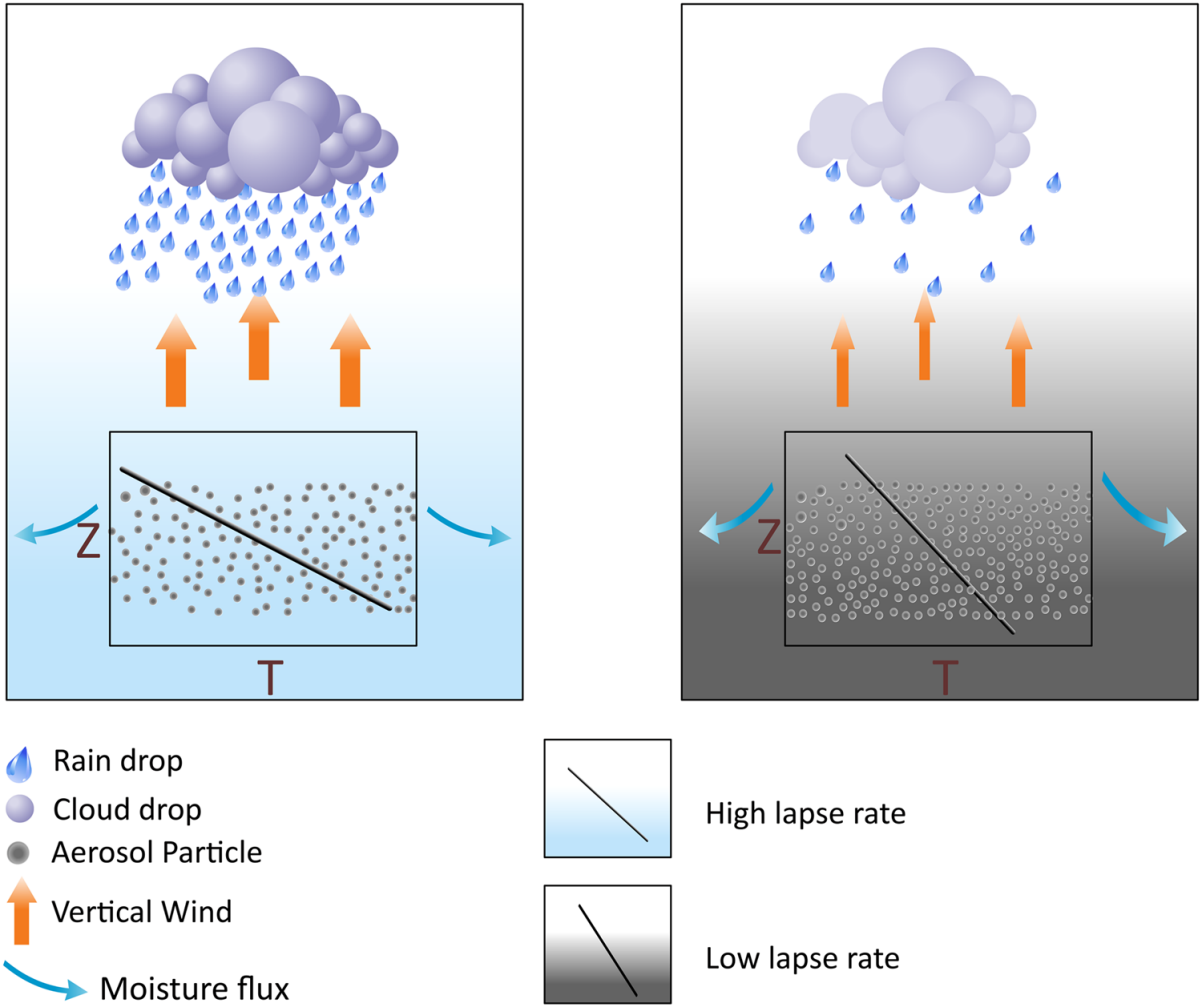
Mechanisms of aerosol induced suppression: Reduced aerosol loading makes the atmosphere unstable causing reduced divergence of moisture and normal convection leading to uplift of moisture and subsequently normal precipitation. While in case of increased aerosol loading the divergence of moisture is enhanced accompanied with stable atmospheric conditions. Conflation of these effects suppresses the precipitation. Figure was created using CorelDRAW × 6 (www.coreldraw.com).

Convection is reported to accommodate positively, on daily to monthly timescales, to radiative effects of absorbing aerosols like black carbon leading to short-term increases in precipitation^8,9^. Specifically, active phases following monsoon break periods, were linked to a build-up of aerosols which caused aerosols moisture convergence and onset of rainfall^47,52^, on time-scales of about 20 days. However, reduction in convection with increases in black carbon aerosols is reported, both in observation studies of biomass burning^40^ and modelling studies^12^. Modeling study conducted by Das *et al*.^53^ reported increase in moisture divergence with increased absorbing aerosols. Guo *et al*.^54^ point out that a significant threshold of black carbon loading is necessary to induce convection, which was found only in simulations using 5xBC of present day emissions. The overall mesoscale mechanism observed here (Fig. 5), is enhancement in aerosol levels causing atmospheric stabilization, with increased horizontal moisture divergence, reduced convection and vertical velocity and subsequent suppression of precipitation. These two effects of limited moisture availability and restricted convection conflate and contribute to the suppression of precipitation.

### Implications for Monsoon Break Spells

Break spells are inherent to the Indian monsoon. However, their intensity and duration plays an important role in determining deficient precipitation or drought conditions^2,55^. While several definitions are used to identify monsoon break spells, a widely accepted one is based on a normalized anomaly threshold of one standard deviation below the mean^2,55,56^, which occurs for at least three consecutive days (Fig. S7).

**Figure 6:**
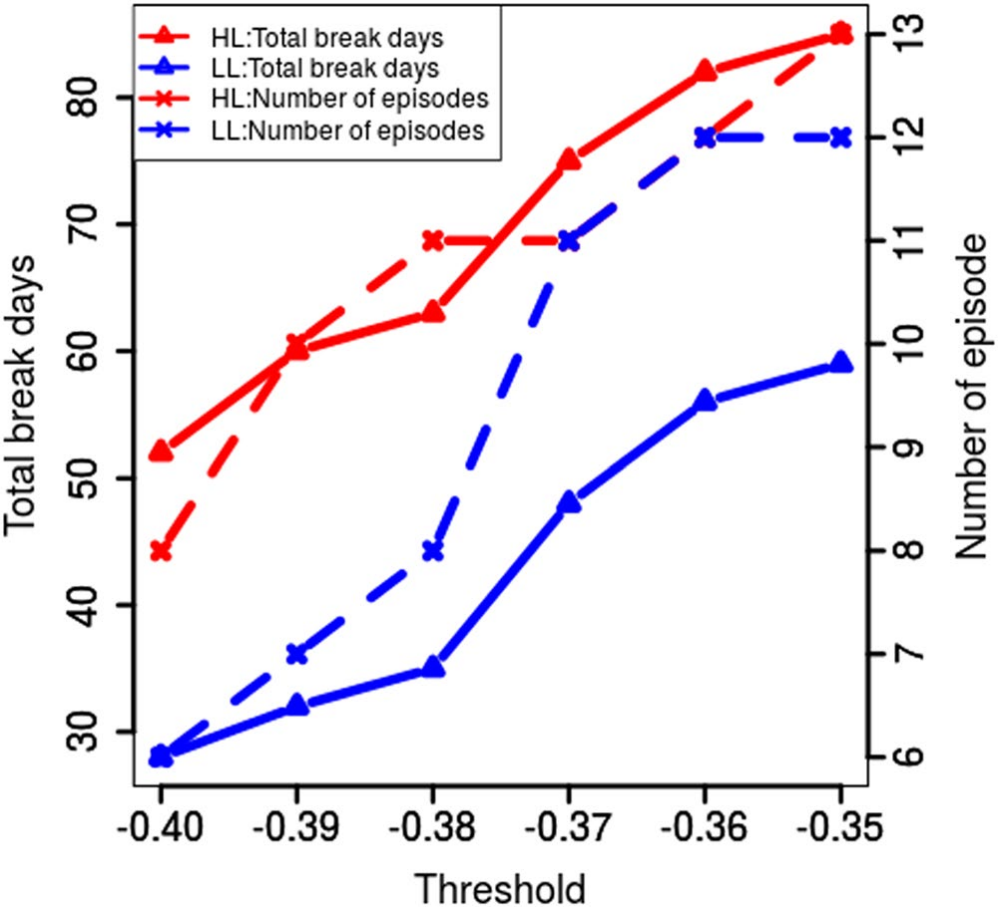
Break characteristics in HL and LL clusters: Total number of break days and frequency of episodes of break spells in HL and LL cluster with varying AOD threshold. Increased number of break days and break episodes were found in regions with increased aerosol levels. Figure was created using R statistical tool v3.3.1 (https://www.r-project.org/).

The possible influence of aerosols on mediating break spells was examined through comparing break occurrence in regions of higher (HL) and lower (LL) aerosols (Fig. 6). A larger number of total break days and increased frequency of break spells and prolonged break spells (lasting seven days or longer) was found in HL regions (3–4 times in a season) than in LL regions (1–2 times in a season). The chosen threshold of precipitation anomalies corresponded to the negative one standard deviation and more stringent decreases (Supplementary Fig. 8) and results were averaged over all threshold values. It is accepted that prolonged or extended breaks (lasting seven days or more) often result in droughts^2,55^. Recent studies examining monsoon variability over India during the last 50 years found an increased duration^57^ and frequency^58^ of break spells. However, explicit attribution to aerosol effects was not investigated in the earlier studies.

The widespread nature of aerosol-induced rainfall suppression observed here, is evidenced by their occurrence not only in 2004 and 2009, widely reported as deficient monsoon years on sub-continental scales^14,35^, but also in 2005, acknowledged as a normal monsoon year. It was suggested that when the Indian summer monsoon anomaly is large (>15%), it is often uniform across the country, but when it is within a few percent of the mean, several regions could have deficit or excess precipitation^1^. This suggests that the aerosol-induced causal mechanisms uncovered here could aggravate break spells and precipitation deficits in normal monsoon years with modest monsoon precipitation anomalies. In other years, where the analysis was inconclusive due to lack of data, the use of a finer resolution or larger dataset could yield better insights.

Intra-seasonal oscillations of the Indian monsoon typically occur on cycles of 6–9 days, 10–20 days, and 30–60 days^59^. Monsoon break periods are generally associated with increased surface pressure anomalies, weaker moisture-laden low-level flows from the southern Indian Ocean, decreased cyclonic vorticity over the monsoon region, and positive anomalies in outgoing longwave radiation associated with the scarcity of clouds^44,55,60^. The aerosol-induced suppression of precipitation seen in this study, which results from mesoscale atmospheric stabilization on shorter time scales of 3–5 days (Fig. 1c), is therefore a distinct phenomenon, whose interplay with monsoon break dynamics warrants further investigation.

## Summary and Discussion

Anthropogenic pollution particles over South Asia comprise a mixture of light scattering species (including sulfate, nitrate, organic carbon and others) and light absorbing black carbon (or soot), the latter emitted primarily by traditional technologies burning biomass fuels^61^. This study uncovered a causal influence of aerosols on repeated meso-scale suppression of precipitation, which occurred throughout the monsoon season. The suppression mechanism was mediated primarily through a radiative pathway acting to increase atmospheric stability and horizontal divergence of moisture.

Consensus in previous studies points to monsoon rainfall weakening linked to an asymmetric inter-hemispheric energy balance change attributed to aerosols^5,7^ and short-term rainfall enhancement linked to radiative effects of non-local absorbing aerosols^8,9,47,52^. In this study, short-term rainfall suppression is linked to radiative effects of coincident aerosols, acting through repeated atmospheric stabilization, reduction in convection and increased moisture divergence, leading to aggravation of monsoon break conditions. Interestingly, in addition to being manifested in deficient monsoon years, causal influences of aerosols on precipitation suppression also occurred in a normal monsoon year, indicating the possibility of a widespread occurrence of this phenomenon.

The causal influence of aerosols on precipitation suppression is relevant to the inter-annual variability of monsoon precipitation and the timing of monsoon break spells. Prolonged and intense breaks in the monsoon were associated with rainfall deficits^55^, which have been linked to reduced food grain production during latter half of the twentieth century^1^. Thus, aerosol-induced precipitation suppression and aggravation of break spells, uncovered here, could influence future rainfall deficits and agricultural vulnerability in India.

## Methods

### Data set

Aerosol and cloud properties from satellite observations^22–24^, precipitation from ground based measurements^25^, and meteorological variables from European Centre for Medium-Range Weather Forecasts (ECMWF) re-analysis (ERA)-interim reanalysis data^26^ from 2000–2009 over the Indian subcontinent (6.5–40° N and 66.5–100° E), at 1 × 1° resolution for the monsoon months of June to September (JJAS), were used for the analysis. Level-3 (L3) atmospheric aerosol data, retrieved from the moderate resolution imaging spectroradiometer (MODIS) on board the Earth Observing System’s (EOS) Terra (MOD08_D3v6) and Aqua (MYD08_D3v6) satellites, made available through the National Aeronautics and Space Administration (NASA) Deep blue (Collection-6) algorithm^24^, were used for aerosol optical depth (AOD). The data includes AOD retrievals over regions with high reflectance. Positive values of absorbing aerosol index (AAI) from the total ozone mapping spectrometers (TOMS) on board the Earth Probe satellites and the ozone monitoring instrument (OMI) sensor on board the EOS Aura satellite, were derived using the Earth Probe TOMS^22^ and OMAERUV^62^ algorithms. These measure absorbing aerosols that are within the range of 0.5 and 3.5 km^63^ and neglect the residues related to purely scattering aerosols^62^. MODIS L3 cloud droplet effective radius (CDER) data, obtained from both EOS Terra (MOD08_v3) and Aqua (MYD08_v3) satellites, were used. Lapse rate was calculated using the temperature of nine layers of atmosphere between 1000 and 750 hPa, corresponding to the lower troposphere in the ERA-interim dataset^26^. Precipitation data, as a gridded product, were obtained through the interpolation of data from 1803 irregularly located meteorological stations over India, with approximately 350 pixels over the Indian domain^25^.

### Data Processing

The description and source of the data is listed in Table S4 for years 2000–2009 at resolution of 1 × 1^o^. The spatial coverage for the study region was 6.5–40 °N to 66.5–100 °E. AOD values less than 0.8 were retained for the analysis as higher values may be due to misclassification of clouds as aerosols^40^. Daily pixel-wise absolute value for each variable was transformed into normalized anomaly. To achieve this, at a given pixel, deviation of daily absolute value from its mean daily value (across years) was divided with standard deviation of daily absolute value (across years). This was repeated for all the pixels. Further, season average anomaly was calculated by taking mean of normalized anomaly for each year and every pixel. Normalized daily anomaly (Δ*x*_*tiy*_) for each variable was calculated as described below:

The normalized anomaly (Δ*x*_*tiy*_) was the deviation of variable for a specific day (*t*) and pixel (*i*) from the mean (calculated across years), normalized by its standard deviation. Further, seasonal average anomaly $(\overline{{\rm{\Delta }}{x}_{iy}})$ was calculated for each year and each pixel. Depending upon the value of $\overline{{\rm{\Delta }}{x}_{iy}}$ the complete 122-day temporal series of anomaly (Δ*x*_*tiy*_) was assigned to either low value cluster or high value cluster.

1 $$\overline{{x}_{ti}}=\frac{\sum _{y=1}^{Y}{x}_{tiy}}{Y},\{i=1,2,\mathrm{...}.,N;t=1,2,\mathrm{...}.,{\rm{T}}\}$$

2 $$\sigma ({x}_{ti})=\sqrt{\frac{\sum _{y=1}^{Y}{({x}_{tiy}-\overline{{x}_{ti}})}^{2}}{Y-1}},\{i=1,2,\mathrm{...}.,N;t=1,2,\mathrm{...}.,{\rm{T}}\}$$

3 $${\rm{\Delta }}{x}_{tiy}=\frac{{x}_{tiy}-\overline{{x}_{ti}}}{\sigma ({x}_{ti})},\{i=1,2,\mathrm{...}.,N;t=1,2,\mathrm{...}.,{\rm{T}};y=1,2,\mathrm{...}.,{\rm{Y}}\}$$

4 $${\overline{{\rm{\Delta }}x}}_{iy}=\frac{\sum _{t=1}^{T}{\rm{\Delta }}{x}_{tiy}}{T},\{i=1,2,\mathrm{...}.,N;y=1,2,\mathrm{...}.,{\rm{Y}}\}$$

5 $${\overline{{\rm{\Delta }}x}}_{iy}=\{\begin{array}{c} > 0,\,i\,{\rm{is}}\,{\rm{an}}\,{\rm{high}}\,{\rm{value}}\,{\rm{pixel}}\,{\rm{in}}\,{\rm{year}}\,y\\  < 0,\,i\,{\rm{is}}\,{\rm{a}}\,{\rm{low}}\,{\rm{value}}\,{\rm{pixel}}\,{\rm{in}}\,{\rm{year}}\,y\end{array},\{i=1,2,\mathrm{...}.,N;y=1,2,\mathrm{...}.,{\rm{Y}}\}$$

here *t* denotes a day in June-September (JJAS) (*t* = 1, 2, …, *T*). Pixels are denoted as *i* (*i* = 1, 2, …, *N*) and *y* denotes years (*y* = 1, 2, …, Y). *T* is total number of days in JJAS, *N* is total number of pixels and *Y* is total number of years.

The season averaged pixel-level AOD and precipitation anomalies were subjected to hierarchical clustering for each of the years (2000–2009). Pixels were assigned to (a) high AOD-low precipitation (HL), (b) low AOD-low precipitation (LL), (c) high AOD-high precipitation (HH), and (d) low AOD-high precipitation (LH) clusters. The high AOD-low precipitation and low AOD-low precipitation clusters were selected to investigate possible effects of different levels of aerosols on precipitation suppression. Clustering process is described below:

Pixels were first clustered into high and low AOD anomaly pixels depending upon $\overline{{\rm{\Delta }}{x}_{iy}}$ AOD values. These pixels were further clustered into high and low precipitation anomaly pixels using $\overline{{\rm{\Delta }}{x}_{iy}}$ precipitation values. Thus, pixels were clustered into four clusters i.e. (a) high AOD-low precipitation (HL), (b) low AOD-low precipitation (LL), (c) high AOD-high precipitation (HH), and (d) low AOD-high precipitation (LL). Once the pixels were assigned to respective clusters, cluster average of all the pixels was taken to get an average temporal series Δ*x*_*ty*_ for each year which was used for causality analysis.

6 $${\rm{\Delta }}{x}_{ty}=\frac{\sum _{i=1}^{{\rm{M}}(y)}{\rm{\Delta }}{x}_{tiy}}{M(y)},$$

where *M*(y) is the number of pixels in a given cluster, *t* is the day and *y* is the year.

HL clusters for years 2004, 2005 and 2009 are shown in Supplementary Fig. 2 while LL clusters are shown in Supplementary Figs 3,4. For year 2004, HL cluster had 113 pixels while LL cluster had 98 pixels while year 2009 had 156 and 169 pixels respectively for HL and LL clusters. HL cluster corresponded to highly deficient region for year 2004 and peninsular regions for 2009, as reported in literature^35,64^. Though 2005 was normal year the HL pixels (belonging to north-eastern region of India) received less precipitation compared to other years^65^. The above clustering and analysis was based on normalized anomaly values. The definition of anomaly ensured that regional effects were excluded while establishing causality.

The individual time series were found to be stationary using Kwiatkowski-Phillips-Schmidt-Shin (KPSS) test or were made stationary by first order differencing, if the original time series was non-stationary. In the cause-effect model, Granger causality (GC) was tested pair-wise both ways and once the causality was established, the lagged correlation coefficient (lag obtained from GC analysis as discussed next) was calculated and provided as input to path analysis. Lags with washout (i.e. precipitation causing AOD, feedback (i.e. AOD causing precipitation and precipitation causing AOD), and lags associated with statistically not significant correlations were excluded from the analysis. The statistical significance tests were performed at α = 0.1 throughout the study.

### Granger causality

The notion of Granger causality (*GC*) was first introduced by Granger^28^. It relies on the principle that the causal event leads its effect and has unique information about the future. A variable *Y* is said to Granger cause variable *X*, if inclusion of past information of both *Y* and *X* gives statistically significant improvement in the prediction of *X* as compared to only inclusion of past information of *X*. *GC* has been applied in climate domain for causal attribution^66–68^.

Consider stationary time series of *Y*_*t*_ and *X*_*t*_ to test the null hypothesis of no Granger causality. Towards this end, an auto-regressive model of *X*_*t*_ is compared with auto- and cross-regressive model of *X*_*t*_ involving *Y*_*t*_ as,

7 $${X}_{t}={\alpha }_{0}+{\alpha }_{1}{X}_{t-1}+\mathrm{.....}.+{\alpha }_{n}{X}_{t-n}+{\varepsilon }_{x}$$

8 $${X}_{t}={\tilde{\alpha }}_{0}+{\tilde{\alpha }}_{1}{X}_{t-1}+\mathrm{.....}.+{\tilde{\alpha }}_{n}{X}_{t-n}+{\beta }_{1}{Y}_{t-1}+\mathrm{.....}.+{\beta }_{n}{Y}_{t-n}+{\varepsilon }_{xy}$$

If the variance of the residual in the second model, labelled ${\sigma }_{{\varepsilon }_{xy}}^{2}$, is significantly less than the variance of the residual in the first model, labelled ${\sigma }_{{\varepsilon }_{x}}^{2}$, then the inclusion of information of *Y* is improving the prediction of *X*_*t*_ implying that *Y* is Granger causing *X*. *GC* was tested at varying lags, and lags with statistically significant causality were retained for further analysis. First order difference was performed for all the variables to ensure stationarity before performing *GC* test for years 2004, 2005 and 2009. Along with this, causality of a particular day was also tested by comparing the regression model with and without inclusion of a particular day value.

9 $${X}_{t}={\alpha }_{0}+{\alpha }_{1}{X}_{t-1}+\mathrm{.....}.+{\alpha }_{n}{X}_{t-n}+{\tilde{\varepsilon }}_{x}$$

10 $${X}_{t}={\tilde{\alpha }}_{0}+{\tilde{\alpha }}_{1}{X}_{t-1}+\mathrm{.....}.+{\tilde{\alpha }}_{n}{X}_{t-n}+{\beta }_{n}{Y}_{t-n}+{\tilde{\varepsilon }}_{xy}$$

If ${{\sigma }^{2}}_{{\tilde{\varepsilon }}_{xy}}$ is significantly less than ${{\sigma }^{2}}_{{\tilde{\varepsilon }}_{x}}$ the inclusion of information of *Y*_*t−n*_ is improving the prediction of *X*_*t*_ implying that *Y*_*t−n*_ (i.e. a particular n^th^ day in the past) is Granger causing *X*. The results found were same as with conventional causality methods.

### Path Analysis

Proposed by Wright^29^, path analysis enables splitting of the net effect of one variable on other variables into direct and indirect effects. The direct effect is the path coefficient of the directed edge between two variables under consideration. The indirect effect is the sum of product of path coefficients for all paths, other than the direct edge, connecting the variables under consideration. To illustrate, consider the example in Supplementary Fig. 9 ^69^ where two exogenous variables Z_1_ and Z_2_ are affecting a common endogenous variable Y. The variables Z_1_ and Z_2_ are correlated with Y. The correlation coefficients ρ_12_, ρ_1y_ and ρ_2y_ are available for a given data set. In Supplementary Fig. 9 the double headed arrow between the exogenous variables Z_1_ and Z_2_ represents the correlation between exogenous variables. The single headed arrow from exogenous variables Z_1_ to Y and Z_2_ to Y signifies that Y is dependent on Z_1_ and Z_2_. The self-loop on variable Y represents the error term and coefficient *p*_*yε*_ represents the error path coefficient. The regression model of Y, in standardized form, can be written as:

11 $$Y={p}_{1y}{Z}_{1}+{p}_{2y}{Z}_{2}+{p}_{y\varepsilon }\varepsilon $$

Path coefficients *p*_*1y*_, *p*_*2y*_ and *p*_*yε*_ are calculated using:

12 $${\rho }_{1y}={p}_{1y}(1)+{p}_{2y}{\rho }_{12}$$

13 $${\rho }_{2y}={p}_{1y}{\rho }_{12}+{p}_{2y}(1)$$

14 $$1={\sigma }_{Y}={p}_{1y}^{2}+{p}_{2y}^{2}+{p}_{y\varepsilon }^{2}+2{\rho }_{12}{p}_{1y}{p}_{2y}$$

Once the path coefficients are calculated the total effect can be segregated into direct and indirect effects. The direct and indirect effects of exogenous variables Z_1_ and Z_2_ on Y are shown in Supplementary Table 5.

In the current work, path analysis was used to segregate the causal influence of aerosol on precipitation into cloud microphysics and radiative pathways. The strength of each path was quantified as product of path-coefficients of edges appearing in that path. The presence (absence) of a statistically significant path coefficient indicates the presence (absence) of the effect.

## Electronic supplementary material

Supplementary information
