# Supplementary material for: Aerosols cause intraseasonal short-term suppression of Indian monsoon rainfall
Source: Sci Rep. 2017 Dec 11;7:17347. doi: 10.1038/s41598-017-17599-1 (PMC5725429; doi:10.1038/s41598-017-17599-1)
Supplement: Supplementary file 1 — Supplementary information [file 41598_2017_17599_MOESM1_ESM.pdf]

Supplementary information for

Aerosols cause intraseasonal short-term suppression of Indian monsoon rainfall

Authors: Prashant Dave[1], Mani Bhushan[1,2], Chandra Venkataraman[*1,2]

Affiliations:

[1] Interdisciplinary Programme in Climate Studies, Indian Institute of Technology Bombay, Mumbai-400076, India

[2] Department of Chemical Engineering, Indian Institute of Technology Bombay, Mumbai-400076, India

*Correspondence to: chandra@iitb.ac.in

This file includes:

Supporting text

Supplementary Figs. 1 to 9

Supplementary Tables 1 to 5

Supporting text:

Overall effect of different pathways

When radiative pathway was excluded from the model (Fig. 3) the unexplained variability in precipitation increased and the increase was of the same order as when column water vapour-precipitation pathway was excluded for years 2004 and 2009 for HL cluster (Table S2).

Effect of surface pressure as common moderator on AOD and VIDMF

In order to investigate the role of surface pressure as a common factor influencing AOD anomaly and VIDMF anomaly in HL cluster, pairwise causality analysis was performed amongst surface pressure, AOD anomaly and VIDMF anomaly. It was found that there is no causal influence of surface pressure anomaly on AOD anomaly. However, statistically significant causal influence of AOD anomaly on surface pressure anomaly was found for years 2004 and 2005. Along with this, causal influence from VIDMF anomaly to surface pressure anomaly was detected for year 2009. The absence of causal influence of surface pressure anomaly on AOD anomaly, rules out the possibility of surface pressure being the driving force behind observed changes in aerosol and subsequent changes in VIDMF. Further, geopotential height anomaly at 1000hPa and 750hPa levels was used as proxy to pressure, results obtained were similar with respect to AOD anomaly i.e. no causal influence of geopotential height anomaly was obtained on AOD anomaly. It supports the hypothesis of changes in VIDMF anomaly driven by changes in aerosols anomaly and not by changes in surface pressure anomaly. Further with varying AOD anomaly threshold it was found that there is increased divergence of moisture and downward wind in HL cluster as evident from cumulative distribution of VIDMF and wind anomalies in HL and LL clusters (Supplementary Fig. 6)

Break analysis

To perform break analysis, precipitation anomaly threshold was identified to define a break period. The identified threshold was found such that it corresponds to the threshold of -1 when precipitation is obtained using area average of data[7]. The cumulative distribution of the precipitation anomaly (Supplementary Fig. 8) for both the approaches were plotted and precipitation threshold anomaly of -0.37 was obtained to define a break spell. Here, to include more stringent threshold criteria as well, a 3 day or longer spell with an anomaly threshold range of -0.37 to -0.4 was examined, corresponding respectively to -1 and -1.2, in terms of normalised anomaly threshold[7]. These varying anomaly thresholds were used to study the

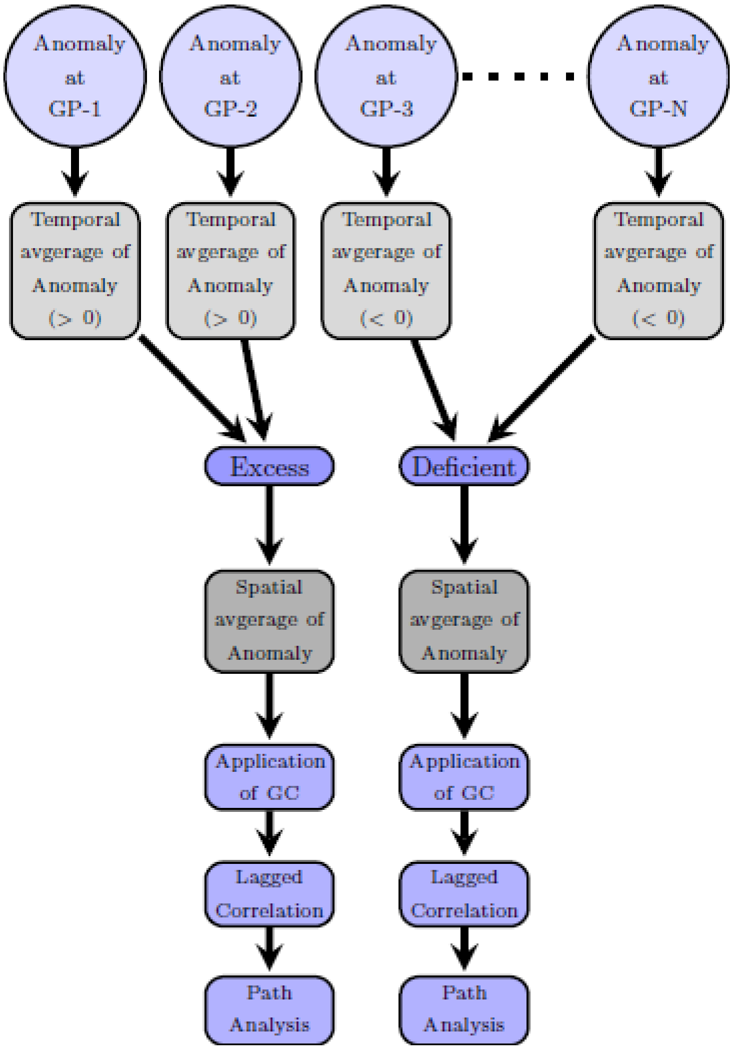

Figure S1. Work flow

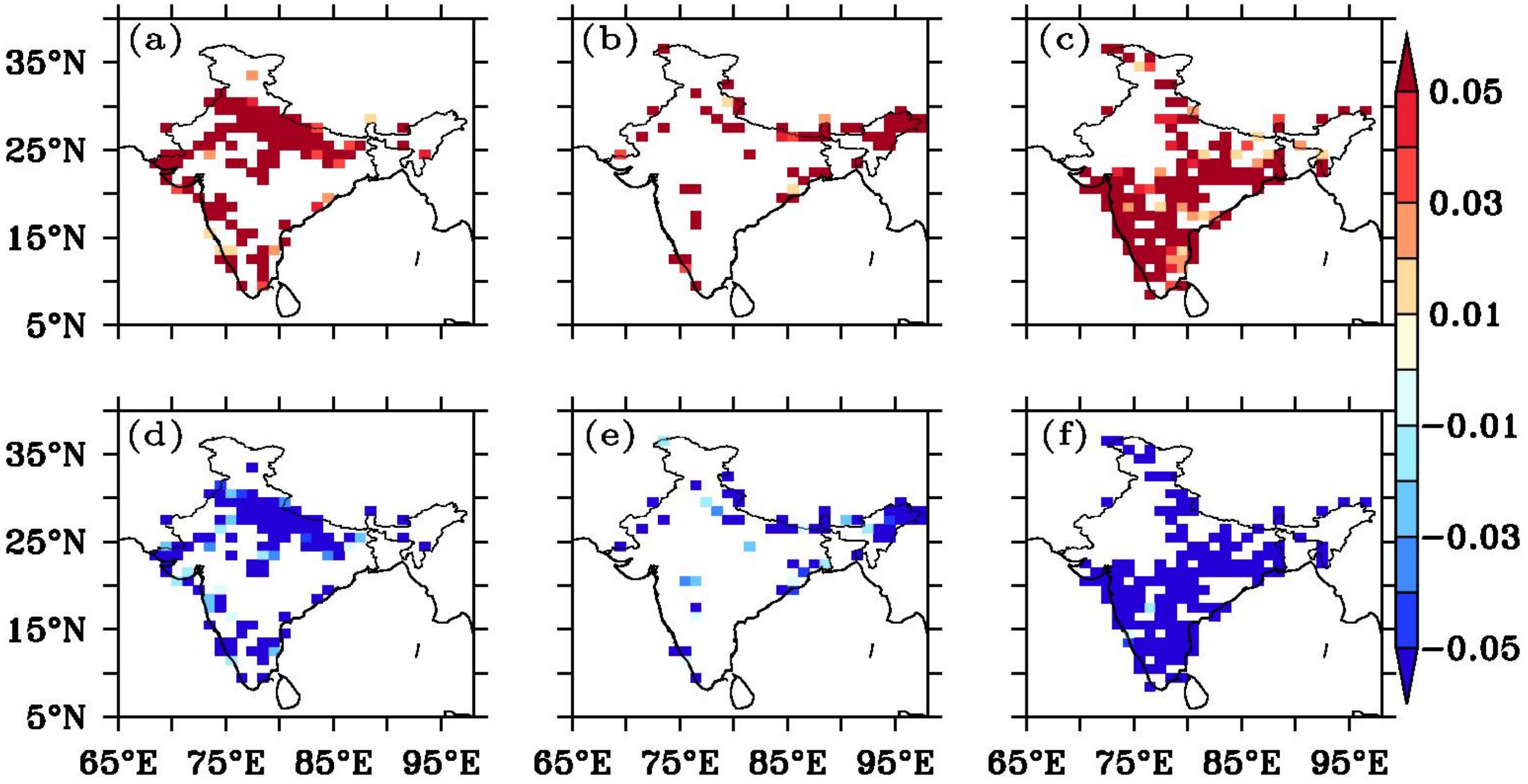

Figure S2. AOD-Precipitation pixels in individual years in HL cluster: (a) 2004 AOD anomaly (b) 2005 AOD anomaly, (c) 2009 AOD anomaly, (d) 2004 Precipitation anomaly, (e) 2005 Precipitation anomaly and (f) 2009 Precipitation anomaly. Figure was created using FERRET v7.0 (http://www.ferret.noaa.gov/Ferret/).

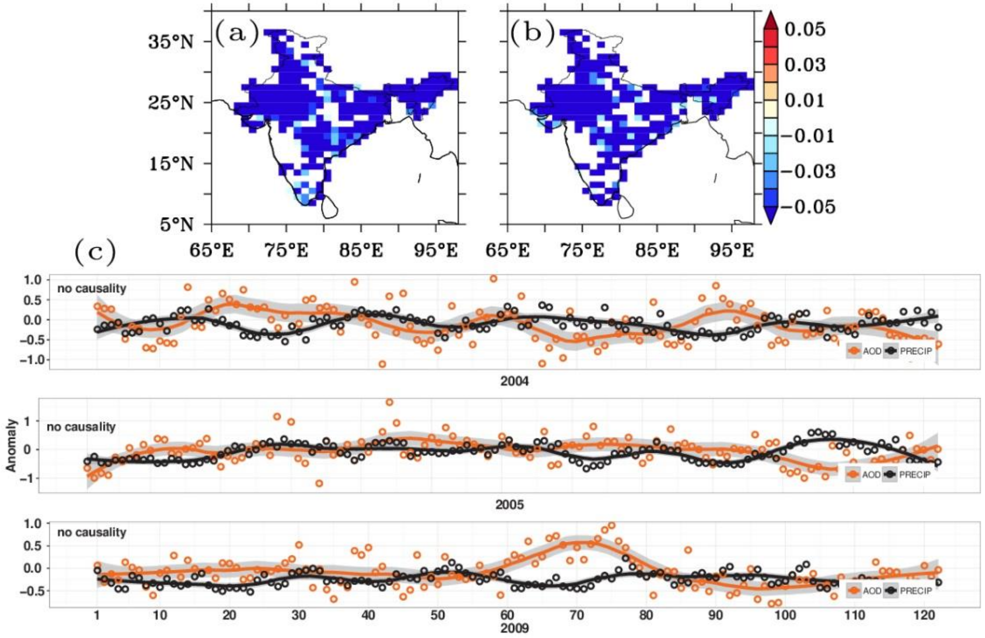

Figure S3. Temporally averaged spatial distribution of LL cluster for 2004, 2005 and 2009 in (a) AOD and (b) Precipitation. (c) Cluster averaged temporal series of AOD and Precipitation. Figure was created using R statistical tool v3.3.1 (https://www.r-project.org/) and FERRET v7.0 (http://www.ferret.noaa.gov/Ferret/)

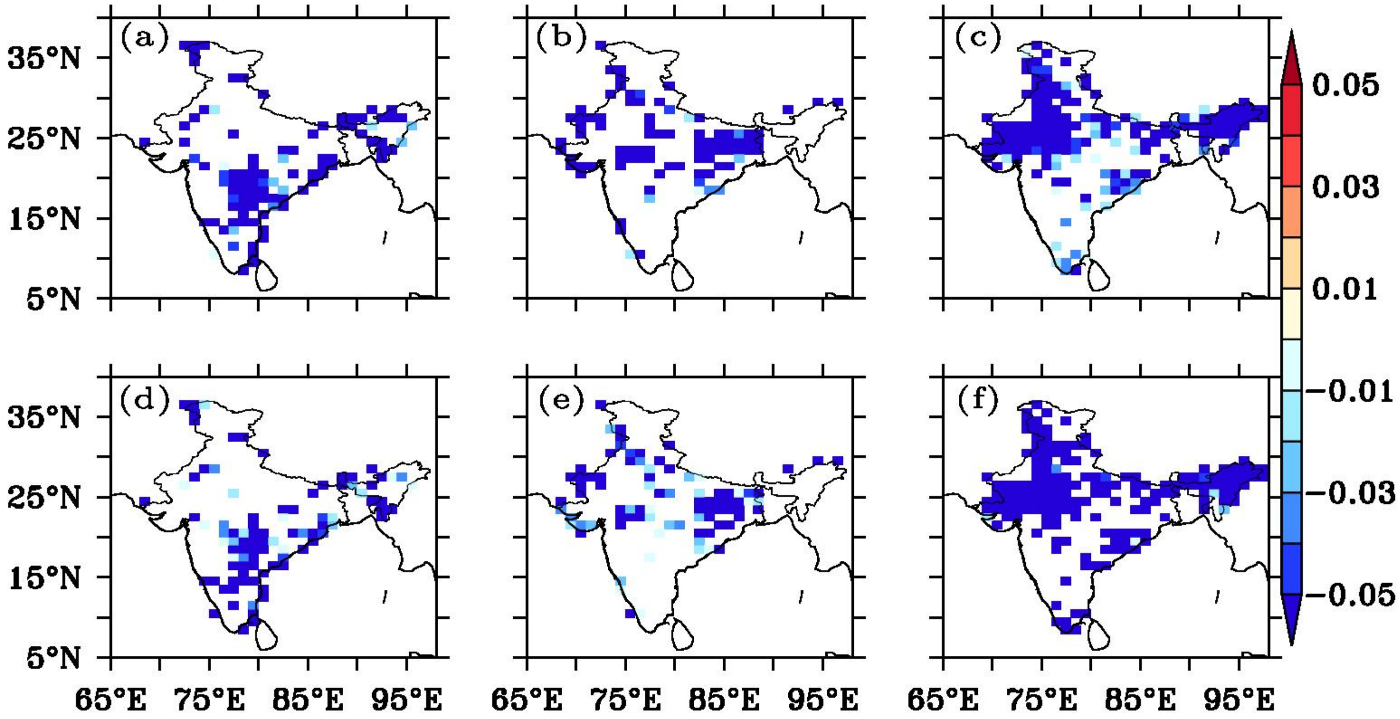

Figure S4. AOD-Precipitation pixels in individual years in LL cluster: (a) 2004 AOD anomaly (b) 2005 AOD anomaly, (c) 2009 AOD anomaly, (d) 2004 Precipitation anomaly, (e) 2005 Precipitation anomaly and (f) 2009 Precipitation anomaly. Figure was created using FERRET v7.0 (http://www.ferret.noaa.gov/Ferret/).

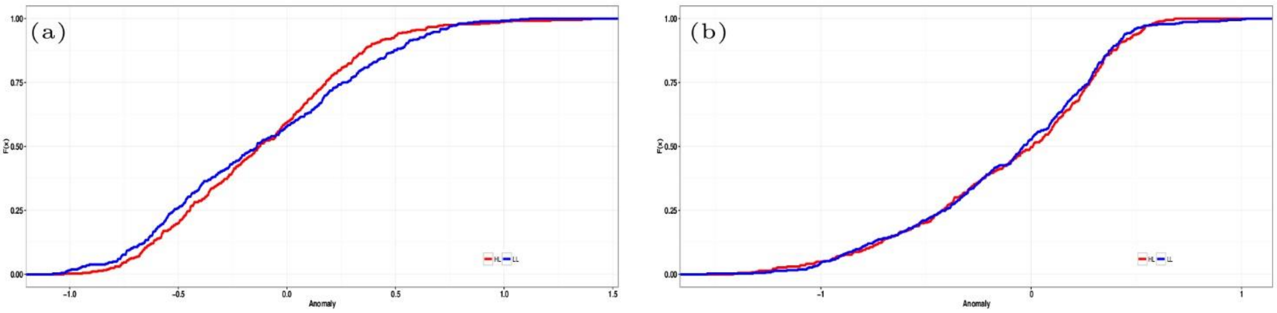

Figure S5. CWV and CF anomaly cumulative distribution for HL and LL clusters. (a) CWV anomaly and (b) CF anomaly. Figure was created using R statistical tool v3.3.1 (https://www.r-project.org/).

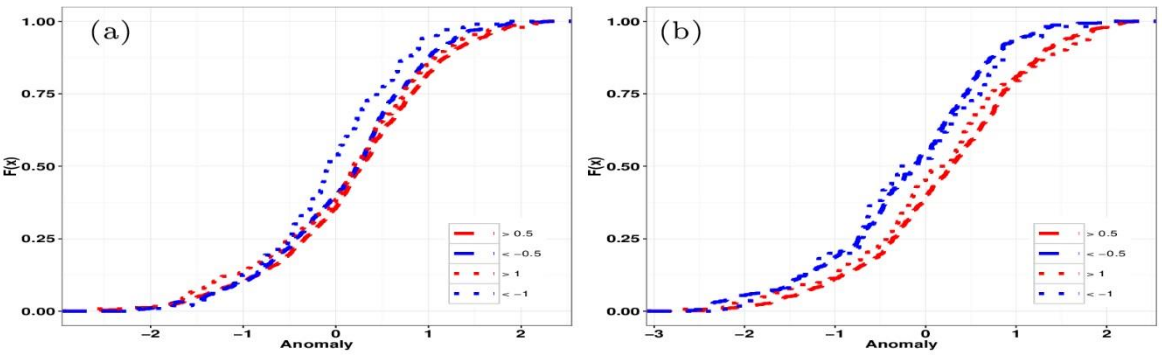

Figure S6. VIDMF and ω850 anomalies with varying AOD threshold. AOD anomaly was kept as ±0.5 and ±1 to select high AOD and low AOD days (consecutive 3-days) legend shows AOD anomaly threshold level. (a) VIDMF anomaly and (b) ω850 anomaly. Figure was created using R statistical tool v3.3.1 (https://www.r-project.org/).

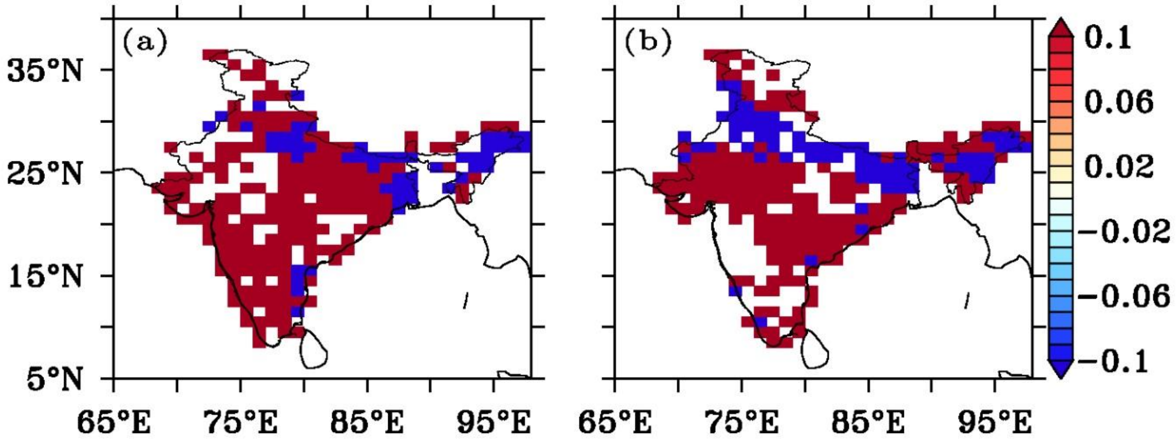

Figure S7. Composite surface pressure anomaly during break days for (a) HL and (b) LL clusters. Figure was created using FERRET v7.0 (http://www.ferret.noaa.gov/Ferret/).

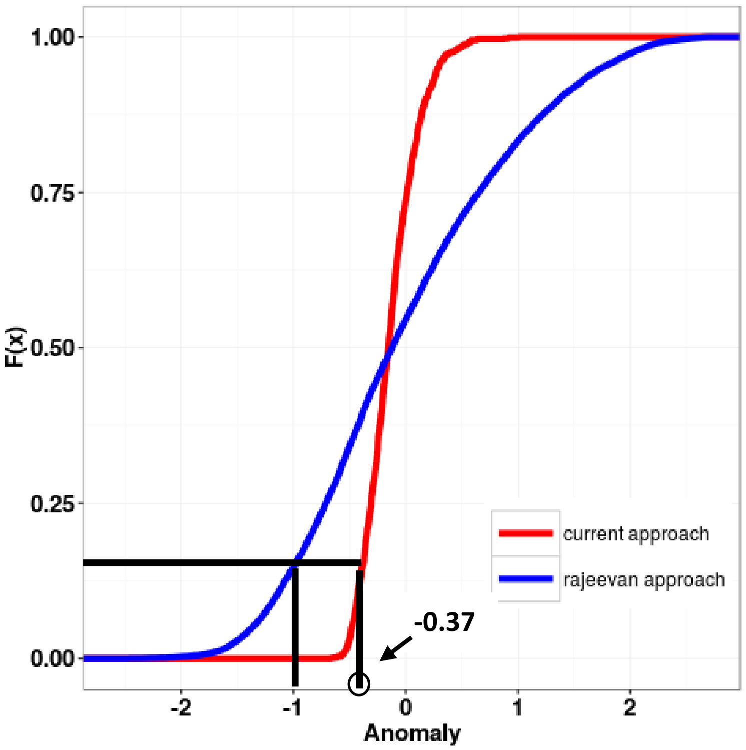

Figure S8. Precipitation cumulative distribution function using area averaged anomaly time series (current approach) and anomaly obtained from area average time series of data[7]. Figure was created using R statistical tool v3.3.1 (https://www.r-project.org/).

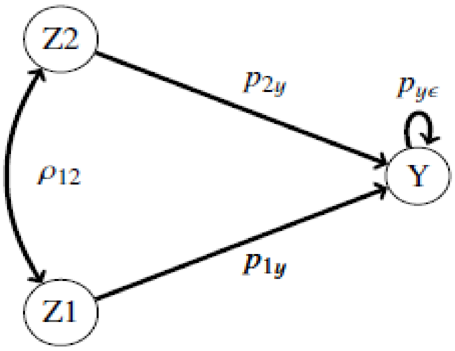

Figure S9: Path analysis example

Table S1. Causal influence of different pathways and precipitation R^2

	HL			LL		
**Pathway**	**2004**	**2005**	**2009**	**2004**	**2005**	**2009**
AOD-CDER-PRECIP	Not significant	-0.035	Not significant	Not significant	Not significant	Not significant
AOD-Lapse rate-PRECIP	-0.172	Not significant	Not significant	Not significant	Not significant	Not significant
AAI-Lapse rate-PRECIP	-0.059	-0.193	-0.064	Not significant	-0.266	-0.065
CWV-CDER-PRECIP	0.169	Not significant	0.182	0.176	Not significant	Not significant
CWV-PRECIP	0.365	Not significant	0.214	Not significant	0.336	0.401
R^2(PRECIP)	0.64	0.38	0.63	0.53	0.58	0.41

Table S2. Precipitation R^2 in different models

	2004		2005		2009	
**Pathways**	**HL**	**LL**	**HL**	**LL**	**HL**	**LL**
Complete Model	0.64	0.53	0.38	0.58	0.63	0.41
Without Lapse rate	0.57	0.43	0.23	0.40	0.57	0.28
Without CDER	0.40	0.37	0.34	0.58	0.46	0.32
Without CWV	0.57	0.52	0.38	0.56	0.50	0.26

Table S3. Surface pressure, AOD and VIDFM causality analysis years 2004, 2005 and 2009 for HL cluster. Lag at which causality exists are listed.

Pathway	2004	2005	2009
Sur. Pressure → AOD	No causality	No causality	No causality
AOD → Sr. Pressure	1-5	2-5	No causality
Sr. Pressure → VIDMF	No causality	No causality	No causality
VIDMF → Sr. Pressure	2	1	2-5
AOD → VIDMF	3-5	1-5	5
VIDMF → AOD	No causality	No causality	2

Table S4. List of variables

**Variable**	**Description**	**Units**	**Source**
AOD	Aerosol optical depth	*Unitless*	MODIS
AAI	Aerosol index	*Unitless*	TOMS:00-04;OMI:05-09
CDER	Liquid cloud droplet effective radius	*µm*	MODIS
CWV	Column water vapour	g/m^2	MODIS
Lapse rate	Lapse Rate	*K/km*	ERA-interim Reanalysis (derived)
ω850	Vertical wind	$Pa\text{-}s^{-1}$	ERA-interim Reanalysis
VIDMF	Vertical integral of divergence of moisture flux	$Kg\text{-}m^{-2}\text{-}s^{-1}$	ERA-interim Reanalysis
SP	Surface pressure	*hPa*	ERA-interim Reanalysis
PRECIP	Precipitation	$mm\text{-}day^{-1}$	IMD-gridded

Table S5. Direct and Indirect effects of exogenous variables on endogenous variable.

	Direct effect	Indirect effect	Net effect
Z_1 on Y	p_{1y}	ρ_{12} x p_{2y}	ρ_{1y}
Z_2 on Y	p_{2y}	ρ_{12} x p_{1y}	ρ_{2y}
